# A micro–computed tomographic analysis of the root canal systems in the permanent mandibular incisors in a Chinese population

**DOI:** 10.1186/s12903-023-02830-5

**Published:** 2023-03-08

**Authors:** Ying Tang, Yihan Wu, Fan Pei, Chao Liu, Yinfeng Qiu, Tao Yang, Yongchun Gu

**Affiliations:** 1 Department of Central Laboratory and Pathology, Ninth People’s Hospital of Suzhou, Soochow University, Suzhou, China; 2 Department of Central Laboratory and Dentistry, Ninth People’s Hospital of Suzhou, Soochow University, Ludang Road 2666#, Wujiang Dist., 215200 Suzhou, China

**Keywords:** Incisor, Dental canal cavity, Root canal preparation, X-Ray microtomography

## Abstract

**Background:**

Comprehensive understanding of the root canal system complexity is critical important for successful root canal therapy. A double root canal system may be present in permanent mandibular incisors with a variable incidence in different ethnic populations. Ignorance or improper management of this canal variation can lead to treatment failure. This in vitro study aimed to identify the anatomic features of root canal systems in the mandibular incisors in a Chinese population by using micro-CT.

**Methods:**

A total of 106 permanent mandibular incisors (53 central incisors and 53 lateral incisors) were collected from a native Chinese population. The teeth were scanned by a micro-CT scanner and then reconstructed three-dimensionally. The canal configurations were detected by Vertucci’s classification, and the number and location of the accessory canals were also identified. The long (*D*) and short diameters (*d*) of the main and accessory canals were measured and *D/d* ratio was calculated at different root levels (cemento-enamel junction [CEJ] level, mid-root level and 1, 2, 3 and 4 mm from the apex). The root canal curvatures in the double-canaled mandibular incisors were measured at the proximal view by using modified Schneider’s method. Chi-square test or Fisher's exact test was used for comparison of occurrence rates. Comparison of means from multiple groups was performed by using one-way ANOVA and LSD post-hoc test.

**Results:**

In regard to the occurrence of double root canals, gender difference was neither detected in the mandibular central (16.0% [male] vs 14.3% [female]; *p* = 0.862), nor in the mandibular lateral incisors (26.9% [male] vs 33.3% [female]; *p* = 0.611). Age group difference was also not detected in the mandibular central (*p* = 0.717) and lateral incisors (*p* = 0.521). The incidence of double root canals was 15.1% (8/53) in the central incisors, and 30.2% (16/53) in the lateral incisors, but the difference did not reach statistical significance (*p* = 0.063). The most frequent non-single canal type was the type III (1–2-1) (18.9% [20/106]), and the other types identified included 1 case of type II (2–1) and 3 cases of type V (1–2). The incidence of accessory canals was 17.9% (19/106), with a mean level of 1.92 ± 1.19 mm from the apex. The frequency of long-oval (2 ≤ *D/d* < 4) and flattened canals (*D/d* ≥ 4), as well as the mean value of *D, d* and *D/d* ratio increased from the apical 1 mm to the apical 4 mm level (the *D/d* ratio increased from 1.9 to 2.9 for the single canals, from 1.4 to 3.3 for the buccal canals and from 1.2 to 2.3 for the lingual canals), and the *D/d* ratio reached the peak at the mid-root level. Double curvatures were detected in 33.3% (8/24) of the buccal canals and 37.5% (9/24) of the lingual canals, and the difference has no statistical significance (*p* = 0.063). The degrees of the primary curvatures were 21.5 ± 7.1 degrees for the buccal and 30.1 ± 9.2 degrees for the lingual canals, and the degrees of secondary curvatures were 27.0 ± 11.4 degrees for the buccal and 30.5 ± 12.5 degrees for the lingual canals in the double curvatures. The degrees of the single curvatures were 14.2 ± 6.3 degrees for the buccal and 15.6 ± 6.0 degrees for the lingual canals. Significant difference was detected among above 6 groups of canal curvatures (*p* = 0.000), and severe curvatures (≥ 20 degrees) were more frequently detected in the double curved canals.

**Conclusions:**

Double-canaled mandibular incisors were not uncommon in the Chinese population, and type 1–2-1 was the most frequent non-single canal type. Gender and age did not significantly impact the occurrence of a second canal in mandibular incisors. Long-oval and flattened canals were very common at different root levels and their incidence increased from apex to the mid-root level. Severe curvatures were frequently detected in the double canal systems, especially in those canals with double curvatures.

## Background

The permanent mandibular incisors are the smallest teeth in the human adult dentition and they commonly possess a single root and a single canal [1]. The roots are wider bucco-lingually than mesiodistally, and usually, a slight longitudinal depression is present on the mesial and/or distal root surface [1]. In some cases, a complex root canal system (mostly double canals) may be present in the root at the bucco-lingual direction, with the incidence ranged from 11 to 45% in different studies, and the discrepancy can be due to the different methodologies or ethnic backgrounds of the subjects [2–13]. The extra canal is prone to be missed diagnosis during endodontic treatment, as the images of the buccal and lingual canals can be overlapped in the conventional 2-dimensional periapical radiograph with standard angles [14]. Ignorance or improper management of this canal variation can lead to treatment failure. Moreover, even when the incisor only has a single canal, the mesio-distally narrow roots are associated with the presence of oval canal or isthmus [15]. The conventional rotary files are inadequate in thorough cleaning and shaping of such canal configurations, and may leave considerable untouched areas in the recesses or irregular canal space [16–18].

There are many in vitro and in vivo methods to evaluate the root canal morphology, which include cross sections, the clearing technique, microscopic investigation, conventional and digital radiography, cone beam computed tomography (CBCT) and micro-computed tomography (micro-CT) [4, 19–21]. Early studies often relied on clearing technique as the gold standard for studying root canal anatomy, but it is destructive to specimens, and can distort internal anatomy and create artifacts [4, 21]. In recent decades, micro-CT has been widely applied on endodontic studies due to its high resolution and nondestruction of the specimens [2, 3]. Supported by built-in or third-party software, it has particular advantages in vividly displaying the complex root canal systems and now has been taken as the golden standard in these kinds of studies [22–24]. The resolution of micro-CT (around 10 μm) is approximately 10-folds higher than that of CBCT (75–400 μm), which offers the ability to visualize those intricate tooth structures, such as root canal variations, accessory canals, isthmus, root resorption, micro-crack formation after instrumentation, etc. [2, 3, 18, 21, 22, 25, 26] It has been well known that the ethnic background and geological regions of the individuals may affect the root and canal variations [27, 28]. Therefore, understanding the root canal morphology of mandibular incisors in the Chinese populations is of great clinical and anthropological significance. There are various classification systems for root canal morphology [29]. Vertucci et al. [4] reported 8 types of configurations according to the pattern of division in the main root canal from leaving the pulp chamber to the root apex, which can describe majority of root canal configurations identified in routine clinical practice or laboratory investigations. By using micro-CT, Leoni et al. [3] studied root canal systems in mandibular incisors of a Brazilian population, and they reported that overall, mandibular central and lateral incisors were similar in terms of the 2- and 3-dimensional (3D) analyzed parameters. Vertucci’s types I (1–1) and III (1–2-1) were the most prevalent canal forms. Wolf et al. [2] performed a similar study based on 125 extracted mandibular incisors from a Germen population, and besides the incidence of different types of the main and accessory canals, they examined the size and shape of the physiological apical foramina, and recommended a final physiological foramen preparation size of ISO 30–35 accordingly.

The purpose of this study was to investigate the morphological features of the root canal system in permanent mandibular incisors in a Chinese population by using micro-CT. Our focus was mainly on the double-canaled mandibular incisors. The distribution pattern of the long-oval and flattened canals at different root levels, as well as the root canal curvature of the double root canal systems, were also analyzed to provide in-depth anatomic information for proper root canal preparation.

## Materials and methods

### Collection of sample teeth

This research project was approved by the Committee in Ethics and the institutional review board (Issuing Number: KY2022-089-01). Extracted permanent mandibular incisors were collected from the Dental Department of the Ninth People’s Hospital of Suzhou, from 2016 to 2022. All subjects were native Chinese, and the teeth were extracted because of periodontal disease, nonrestorable caries, trauma, or prosthodontic reasons. The teeth type (the permanent mandibular central and lateral incisors) was accurately identified by the operator according to its external anatomy, position in the dental arch, tooth sockets in jaw bone, and dental history, and the age of the subject was also recorded. The exclusion criteria were as follows: (a) teeth with a fracture or other major defects in the roots, (b) teeth with root canal fillings, crown restorations, and open apices, and (c) the tooth type cannot be determined correctly. A total of 53 mandibular central and 53 mandibular lateral incisors were included in the current study. The age of the subjects ranged from 14 to 84 years old (mean age = 56.3 ± 16.7 years).

Before investigation, the specimens were immersed in 5% sodium hypochlorite solution for 2 h to remove attached soft tissue. Calculus and stains were removed by an ultrasonic dental scaler. Then the teeth were stored in 10% neutral formalin fix solution.

### Micro-CT scanning

Each incisor was scanned along the tooth axis using micro-CT scanning (SkyScan1174; Bruker-micro-CT, Kontich, Belgium) at a 9-μm voxel size, 800 mA, rotation step of 0.7˚, 50 kVp and filter of 1 mm of aluminum, 1 frame averaging and arch rotation of 180˚.

### Observation of the root canal systems

The micro-CT data sets were then transferred to the Mimics 21.0 (Materialise, Leuven, Belgium) software to perform 3D reconstruction of the teeth and root canal systems. The root canal configurations in the mandibular incisors were examined and described by the Vertucci’s classification [4]: Type I: a single canal is present from the pulp chamber to the apex (type 1–1). Type II: two separate canals leave the pulp chamber but join to form one canal short of the apex (type 2–1). Type III: one canal leaves the pulp chamber and divides into two within the root, but they merge again to exit as one canal (type 1–2-1). Type IV: two separate and distinct canals are present from the pulp chamber to the apex (type 2–2). Type V: one canal leaves the pulp chamber which divides into two separate and distinct canals with separate apical foramina (type 1–2). Type VI: two separate canals join within the root to form one canal, which divides into two distinct canals again short of the apex (type 2–1-2). Type VII: one canal leaves the pulp chamber, divides and rejoins within the root body, and finally redivides into two distinct canals short of the apex (type 1–2-1–2). Type VIII: three separate and distinct canals are present from the pulp chamber to the apex (type 3–3).

The type and number of the accessory canals were also determined.

The calibration was performed by an expert endodontist (*Yongchun Gu*) and an observer (*Ying Tang*). In the pilot study, the observer was trained and calibrated to read the micro-CT images with a sample size of 20 (10 single- and 10 double-canaled incisors) that did not belong to the study sample. The observer evaluated the micro-CT images using sagittal, axial, and coronal views and digital 3D tooth models to identify the root canal morphology, and each tooth received a single score. Disagreements were discussed, until a consensus was reached after adequate deliberation.

The inter- and intra-observer errors was evaluated according to Cohen's kappa test. Each observer evaluated the same 20 teeth twice independently with an interval of two weeks. Substantial Kappa values were obtained (the intra-observer kappa value was 1.0 for both observers [*Yongchun Gu* and *Ying Tang*], and the inter-observer kappa value was 0.9, all *p* = 0.000), suggesting the inter- and intra-observer agreement were both excellent.

### Odontometric analysis

**Fig. 1:**
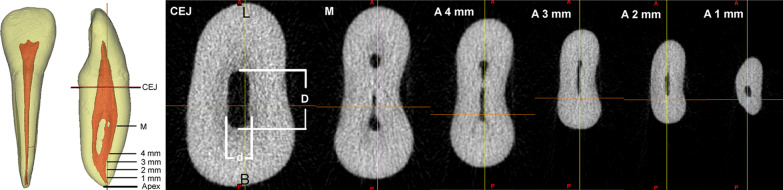
Measurement of the long (D) and short (d) diameter of the root canal at different root levels of a mandibular incisor. *CEJ* is cemento-enamel junction, *M* is mid-root level

The cemento-enamel junction (CEJ) plane was set at the lowest level of CEJ. The apical level was at 1, 2, 3 and 4 mm from the apex, and the mid-root level equally divided the root between the CEJ plane and apex. The vertical distance from the apex to CEJ was measured as the root length. The long (*D*) and short diameters (*d*) of the buccal, lingual, and the single main canal were measured at the level of CEJ, mid-root, and 1, 2, 3 and 4 mm from the apex (Fig. 1). The ratio of *D/d* was calculated, which reflected the degree of deviation from a round canal to a flat canal. The cross-sectional canal shapes can be divided into 3 types according to the *D/d* ratio: round or oval (*D/d* ratio < 2), long-oval (2 ≤ *D/d* ratio < 4), and flattened (*D/d* ratio ≥ 4) [16]. To measure the diameters of the accessory canals, a cross section was performed at the midpoint of the accessory canal, and the *D* and *d* were measured after full amplification of the image by zooming. The vertical length between the divergence site of the accessory canal from the main canal and the apex was measured as the level of an accessory canal.

**Fig. 2:**
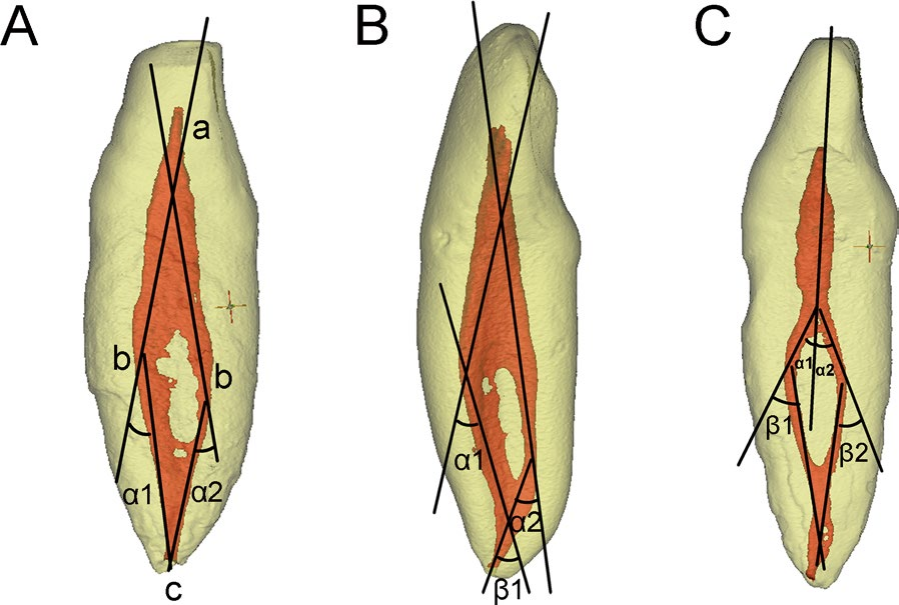
Measurement of the root canal curvatures in double root canal systems in the mandibular incisors (modified Schneider method). *α* is angle of primary curvature; *β* is angle of secondary curvature. **A** Both the buccal and lingual canal exhibit a single curvature. **B** The buccal canal exhibits double curvatures. **C** Both the buccal and lingual canal exhibit double curvatures

Measurements of the root canal curvatures at the proximal view were performed via software Image-Pro Plus 6.0 (Media Cybernetics, Silver Spring, MD) as described previously [30, 31]^.^. Modified Schneider’s method [32] was used to measure the degrees of canal curvatures. Briefly, point *a* was set at the pulp horn. Then, from point *a,* a line was drawn parallel to buccal/lingual wall of the pulp cavity (pulp chamber and the buccal/lingual canal) to point *b*, which was the starting point of the initial curvature (line *ab* can be regarded as the straight access line to the buccal or lingual canal). Point *c* was set at the apical foreman, and the acute angle between line *ab* and line *bc* was measured as Schneider angle of a root canal curvature (Fig. 2). The canal curvatures were classified into three groups based on degrees of the angles: straight (10 degrees or less), moderate (10–20 degrees), and severe (20 degrees or more).

All odontometric measurements were performed by one examiner (*Ying Tang*). Intra-observer agreement was estimated using 10 micro-CT images of double canaled mandibular incisors. The root curvatures were measured twice with an interval of 2 weeks. The intraclass correlation coefficients (ICC) index from a one-way random effects model was calculated. The ICC for intra-observer agreement was 0.943 (95% CI: 0.875, 0.974) (*p* = 0.000), indicating that the measured results were reliable and the error could be ignored.

To evaluate the influence of the gender and age, the sample teeth were divided as male (*n* = 51) or female (*n* = 55) based on gender, and divided into 3 groups based on age (≤ 30 [*n* = 10], 31–60 [*n* = 48], and ≥ 61 yrs. [*n* = 48]).The incidence of double canal systems in different gender and age groups were calculated and compared.

### Statistical analysis

The SPSS 17.0 software (SPSS, Chicago, IL) was used for statistical analyses. Independent student-*t* test was used for comparison of the means between the lateral and central incisors, and Chi-square test or Fisher's exact test was used for comparison of occurrence rates. Comparison of means from multiple groups was performed by using one-way ANOVA and LSD post-hoc test. Student’s *t*-test was used to compare the means between 2 groups. *p* < 0.05 was considered as statistically significant.

## Results

**Fig. 3:**
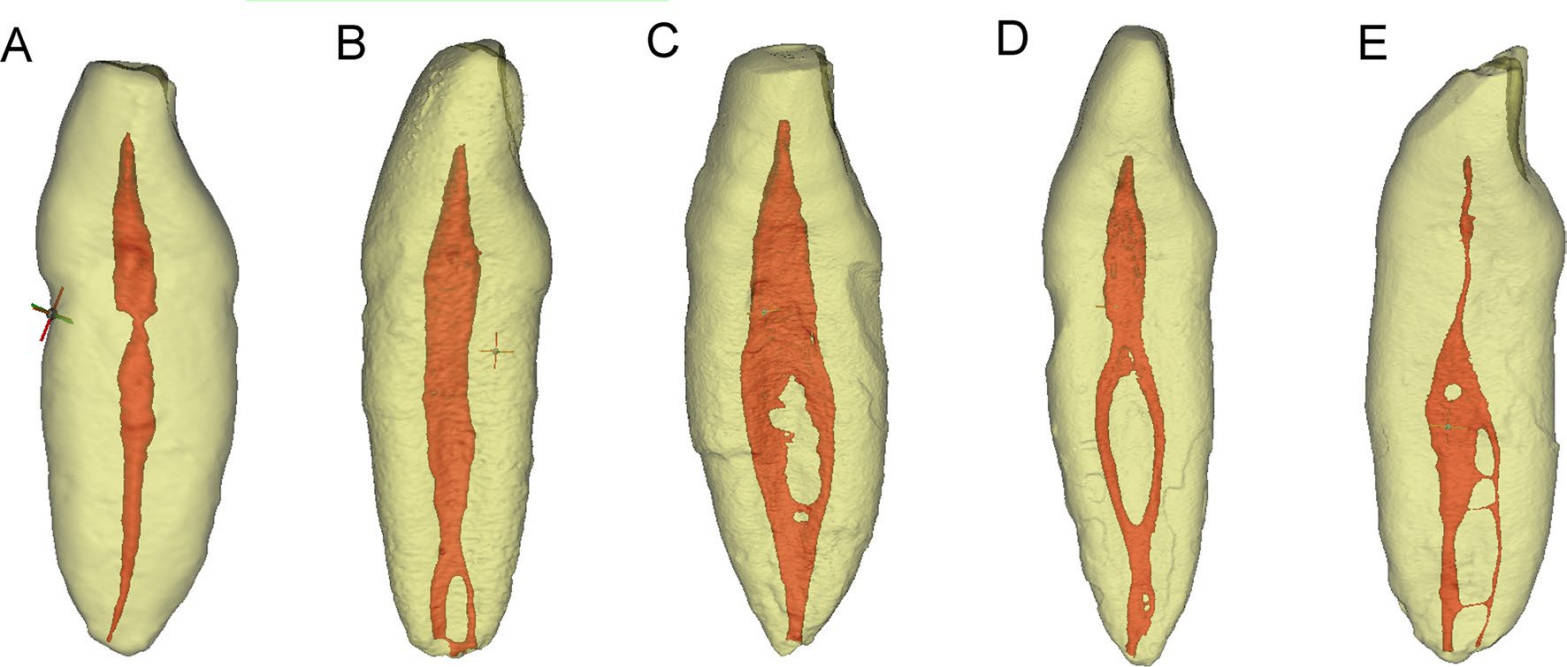
Representative micro-CT 3D images of different types of root canal configurations in mandibular incisors. **A** Type 1–1 canal (the pulp room and root canal can be discriminated), **B** type 1–1 canal with an apical accessory canal (the pulp room and root canal walls are continuous and the boundary between them cannot be discriminated), **C** type 2–1 canal, **D** type 1–2-1 canal, **E** type 1–2 canal with transverse connection canal

The occurrence of single and double root canals in 106 mandibular incisors for both genders were summarized in Table 1. The gender difference was neither detected in the mandibular central incisors (16.0% [4/25] vs 14.3% [4/28]; *X*^2^ = 0.030, *p* = 0.862), nor in the mandibular lateral incisors (26.9% [7/26] vs 33.3% [9/27]; *X*^2^ = 0.258, *p* = 0.611). Fisher’s exact test indicated that the age group difference was not statistically significant in either the mandibular central (*p* = 0.717) or lateral incisors (*p* = 0.521) (Table 2). The root canal configurations in the mandibular incisors were summarized in Table 3, and Fig. 3 shows representative 3D micro-CT images of different root canal configurations. The incidence of double canals was 15.1% in the central incisors, and 30.2% in the lateral incisors, but the difference did not reach statistical significance (*X*^2^ = 3.447, *p* = 0.063). Majority of the non-single canals were type 1–2-1 (91.8%, 21/25), and only 1 case of type 2–1 and 3 cases of type 1–2 canals were detected. We have not detected other complex root canal forms among all 106 sample teeth.

**Table 1:** Root canal number of mandibular incisors in both genders *n*

Gender	*N*	Central incisors (*n* = 53)		Lateral incisors (*n* = 53)		Total incisors (*n* = 106)	
		Single	Double	Single	Double	Single	Double
Male	51	21	4	19	7	40	11
Female	55	24	4	18	9	42	13
Total	106	45	8	37	16	82	24

Gender difference has no statistical significance for each tooth type (*p* = 0.862 and 0.611 for mandibular central and lateral incisors, respectively)

*Single*: single canal; *Double*: double canals

**Table 2:** Root canal number of mandibular incisors in different age groups *n*

Age	*N*	Central incisors (*n* = 53)		Lateral incisors (*n* = 53)		Total incisors (*n* = 106)	
		Single	Double	Single	Double	Single	Double
< 30 yrs	10	3	1	3	3	6	4
30 ~ 60	48	25	4	14	5	39	9
> 60	48	17	3	20	8	37	11
Total	106	45	8	37	16	82	24

Age group difference has no statistical significance for each tooth type (*p* = 0.717 and 0.521 for mandibular central and lateral incisors, respectively)

*Single*: Single canal, *Double*: Double canals

**Table 3:** Root canal configuration of mandibular incisors (Vertucci’s classification) *n* (%)

Type of root canal configuration	Central incisors	Lateral incisors	Total incisors
Type I (1–1)	45 (84.9%)	37 (69.8%)	82 (77.4%)
Type II (2–1)	1 (1.9%)	0 (0.0%)	1 (0.9%)
Type III (1–2-1)	6 (11.3%)	14 (26.4%)	20 (18.9%)
Type V (1–2)	1 (1.9%)	2 (3.8%)	3 (2.8%)
Total	53 (100.0%)	53 (100.0%)	106 (100.0%)

The distribution of the accessory canals (lateral canals and apical deltas) was given in Table 4. The total incidence of accessory canals was only 17.9% (19/106), and there was no statistical difference between the central and lateral incisors (17.0 vs 18.9%, *p* > 0.05).

**Table 4:** Distribution of accessory canals in mandibular incisors (*N*[*n*])

	Central incisors	Lateral incisors	Total Incisors
Lateral canal	3 (4)	1 (1)	4 (5)
Apical delta	7(8)	9 (13)	16 (21)
Accessory canal	9(12)	10 (14)	19 (26)
Main canal only	44	43	87
Total	53	53	106

*N* Number of teeth; *n* Number of accessory canals

**Fig. 4:**
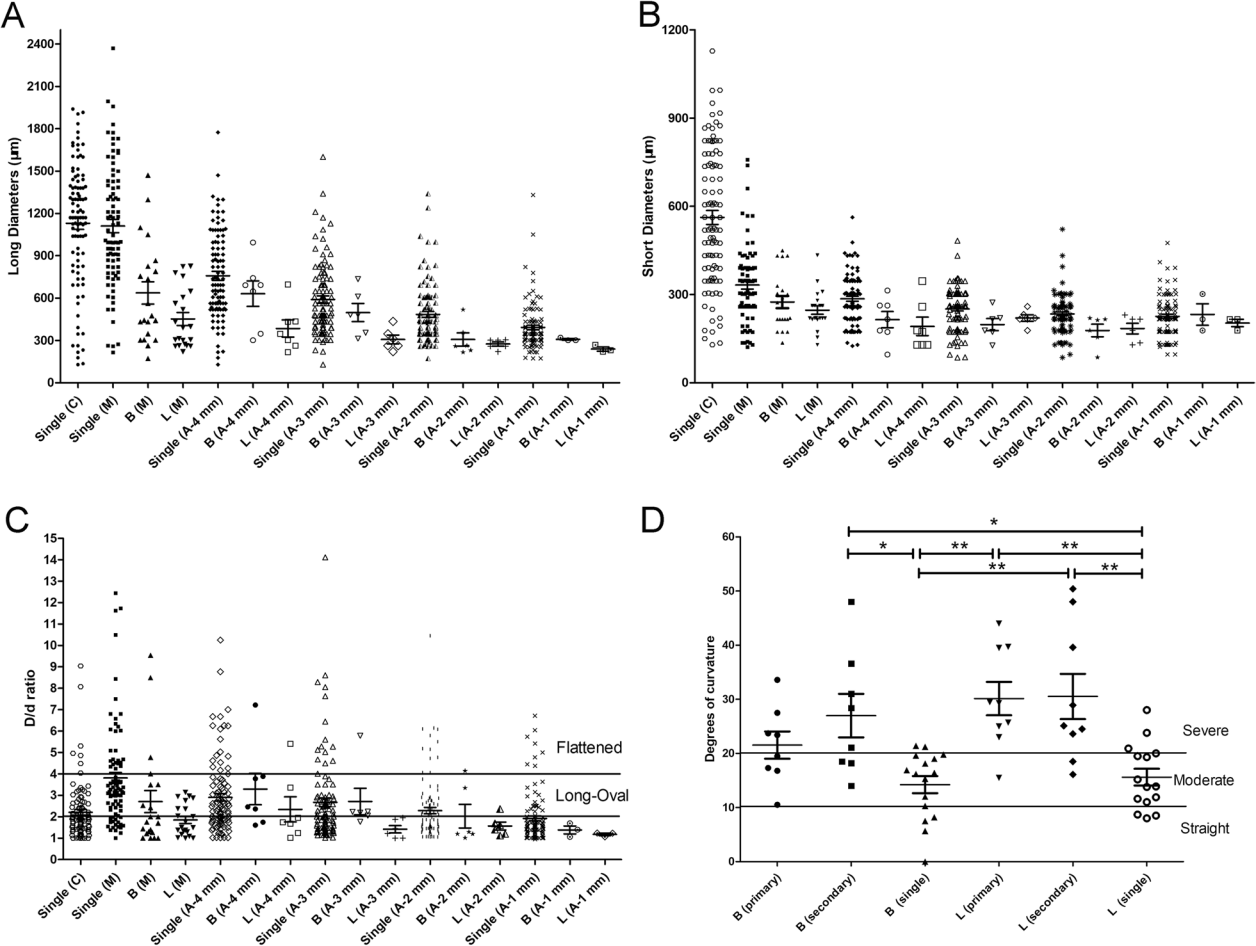
Odontometric measurement results of the root canals in mandibular incisors. **A** Measurement results of long diameters (*D*) at different root levels; **B** measurement results of short diameters (*d*) at different root levels; **C** *D/d* ratio at different root levels;** D** measurement results of root canal curvatures of double-canaled mandibular incisors at the proximal view. Single means the canal display a single curvature, and as the canal display a “s” -shaped double curvatures, the primary and secondary curvature were measured respectively. Error bar is SEM, *p < 0.05, **p < 0.01

The mean root length of mandibular central and lateral incisors was (11.09 ± 0.88) mm, and (12.37 ± 1.24) mm, respectively. The discrepancy has statistical significance (*t* = 6.124, *p* = 0.000). The measurement results of cross-sectional canal diameters (*D* and *d*) at different root levels were shown in Table 5 and Fig. 4A–C. The long diameter was mostly at the bucco-lingual direction, exhibiting a more disperse trend than the short diameter (frequently at the mesio-distal direction). The mean *D/d* ratio, as well as the frequency of long-oval and flattened canals, increased from the apical 1 mm to 4 mm level (the *D*/*d* ratio increased from 1.9 to 2.9 for the single main canal, from 1.4 to 3.3 for the buccal canal and from 1.2 to 2.3 for the lingual canal, and the incidence of long-oval and flattened canals increase accordingly, reaching 44.9% and 19.4% at the apical 4 mm level (for the single canals), respectively. The mean *D* and *d* of the accessory canals (*n* = 26) was 192 ± 107 μm and 131 ± 53 μm, respectively, and the mean *D/d* ratio was 1.43 ± 0.48. Most of them (92.3% [24/26]) were located at the apex 3 mm, with a mean level of 1.92 ± 1.19 mm from the apex.

**Table 5:** Measurement of the long and short diameters of the root canals at different root levels

Root Level	*n*	Canal	Diameter			Degree of roundness *n* (%)		
			*D* (μm)	*d* (μm)	*D*/*d*	*D/d* < 2	Long-oval (2 ≤ *D/d* < 4)	Flattened (*D/d* ≥ 4)
CEJ level	97*	Single	1130 ± 436	562 ± 230	2.2 ± 1.3	57 (58.8%)	33 (34.0%)	7(7.2%)
Mid-root level	84^+^	Single	1111 ± 432	330 ± 128	4.0 ± 3.0	18 (21.4%)	39 (46.4%)	27 (32.1%)
	21	Buccal	616 ± 358	267 ± 90	2.7 ± 2.4	11 (52.4%)	6 (28.6%)	4 (19.0%)
	21	Lingual	447 ± 214	238 ± 58	1.9 ± 0.7	12 (57.1)	9 (42.9%)	0 (0.0%)
4 mm from apex	98^a^	Single	757 ± 312	284 ± 82	2.9 ± 1.7	35 (35.7%)	44 (44.9%)	19 (19.4%)
	7	Buccal	631 ± 238	215 ± 73	3.3 ± 1.9	2 (28.6%)	4 (57.1%)	1 (14.3%)
	7	Lingual	385 ± 160	192 ± 82	2.3 ± 1.5	5 (71.4%)	1 (14.3%)	1 (14.3%)
3 mm from apex	100	Single	590 ± 260	251 ± 71	2.7 ± 2.0	47 (47.0%)	38 (38.0%)	15(15.0%)
	6	Buccal	497 ± 157	198 ± 50	2.7 ± 1.5	1(16.7%)	4(66.7%)	1(16.7%)
	6	Lingual	307 ± 77	220 ± 27	1.4 ± 0.4	6 (100.0%)	0 (0.0%)	0 (0.0%)
2 mm from apex	100	Single	484 ± 209	234 ± 64	2.3 ± 1.5	57 (57.0%)	31 (31.0%)	12 (12.0%)
	6	Buccal	307 ± 115	178 ± 52	2.0 ± 1.4	4 (66.6%)	1 (16.7%)	1 (16.7%)
	6	Lingual	275 ± 34	184 ± 44	1.6 ± 0.5	5 (83.3%)	1 (16.7%)	0 (0.0%)
1 mm from apex	103	Single	392 ± 169	226 ± 70	1.9 ± 1.2	76 (73.8%)	19 (18.4%)	8 (7.8%)
	3	Buccal	308 ± 10	232 ± 63	1.4 ± 0.3	3 (100.0%)	0 (0.0%)	0 (0.0%)
	3	Lingual	239 ± 25	203 ± 22	1.2 ± 0.1	3 (100.0%)	0 (0.0%)	0 (0.0%)

*D*: long diameter; *d*: short diameter. *:9 cases of calcified canal; ^+^:1 case of calcified canal; ^a^:1 case of calcified canal. A calcified canal means at the current root level, the root canal is totally calcified, and no canal space can be detected

The measurement data of canal curvatures in double-canaled mandibular incisors were summarized in Table 6 and Fig 4D. Double curvatures were detected in 33.3% (8/24) of the buccal canals and 37.5% (9/24) of the lingual canals. Significant difference (*F* = 8.265, *p* = 0.000) was detected in mean degrees of Schneider angle among 6 groups of canal curvatures in Table 6. Except the primary curvature of the buccal canals, the degrees of the double curvatures are significantly (all *p* < 0.05) greater than those of the single curvatures, and majority of the double curvatures were severe curvatures, while for single curvatures, most of them were classified as moderate or straight category (Fig 4D).

**Table 6:** Measurement results of root canal curvatures of double-canaled mandibular incisors *n* = 24, (degrees)

Root canal	Type of curvature	*n*	Primary curvature	Secondary curvature
Bucal canal	Double-curved canals	8	21.5 ± 7.1	27.0 ± 11.4
	Single-curved canals	16	14.2 ± 6.3	
Lingual canal	Double-curved canals	9	30.1 ± 9.2	30.5 ± 12.5
	Single-curved canals	15	15.6 ± 6.0	

## Discussion

Micro-CT has proven to be a valuable tool in a wide variety of dental researches [2, 3, 18, 21–26]. Its principal merits include accuracy, repeatability and non-destruction nature, though micro-CT has the demerits of long scan time and elevated radiation exposure, which limit its application in clinical practices [21]. Today, CBCT is the gold standard in clinical examination of the root canal anatomy [10–13, 15, 28], but the voxel size of micro-CT is much smaller than that of the CBCT, which allow for more accurate qualitative and quantitative analysis of the complex root canal systems in the mandibular incisors.

Information about the distribution pattern of root canal variations in mandibular incisors may be useful for clinicians to predict the probability of the occurrence of a second canal, and set a more efficient treatment plan. Previous studies have demonstrated that gender and age both affected the root canal morphology [20, 33, 34]. However, in this micro-CT study, the gender and age-group difference in occurrence of double canals were not detected. A recent CBCT study [33] in a Saudi subpopulation showed the prevalence of canal variations in mandibular canine was higher in females than in males, but gender difference was not detected in mandibular incisors, which is in agree with our findings; moreover, the authors reported that the younger patients showed more variations than the older patients in terms of maxillary laterals, mandibular central, laterals and canines, respectively. Another CBCT study by Karobari et al. [20] demonstrated that in a Malaysian population, the prevalence of canal variations in mandibular incisors was higher in males and the age group of 20–30 years than in females and other age groups (*p* < 0.001). The Malaysians showed significant more canal variations in mandibular incisors than Chinese, and the discrepancy may be due to the different methodologies, ethnicities and sample size [20]. As shown in Table 3, the incidence of double canals was 15.1% and 30.2% in the mandibular central and lateral incisors, respectively. The latter was exactly 2-folds greater than the former. Therefore, clinicians should be doubly careful to avoid missing the second canal during treatment of the mandibular lateral incisors. Our data are much lower than those reported by Leoni et al. [3] in the Brazilian population (the incidence of non-single canals was 50% and 38% in the mandibular central and lateral incisors, respectively), and also lower than that in the German population reported by Wolf et al. [2] (the incidence of non-single canal was 44.0% among all mandibular incisors, as compared to 23.6% in our study). However, our results are higher than those reported by Lin et al. [34] in a Taiwanese population, in which the incidence of 2 canals was 10.9% and 25.5% for the mandibular central and lateral incisors, respectively.

In regard of the types of root canal configuration, there are many classification systems with their own distinct advantages, as well as limitations [29]. Vertucci’s classification included majority of complex configurations, and therefore, were one of the most commonly used classification systems [4, 29]. However, some observers may feel confused over how to define inter-canal communications, which can either be regarded as an integral part of the root canal configuration with an impact on the canal classification, or simply as a minor feature with no impact on the classification [35]. In this study, the inter-canal communications have not posed much difficulty in the canal classification, and we has only detected 3 types of non-single canals (21 cases of type III [1–2-1], 3 cases of type V [1, 2], and 1 cases of type V [2–1]). The type 1–2-1 is the most frequent variation form (accounting for 18.9% of total specimens, and 91.8% of specimens with non-single canals), which is in agreement with the previous report [2, 3, 15]. However, Leoni et al. [3] and Wolf et al. [2] described 9 and 8 new types of complex configurations in their sample teeth, respectively, suggesting that the root canal systems in mandibular incisors of the Chinese subjects were relatively less complex than those reported in other ethnic populations, which is favorable for endodontic treatment. In regard to the type 2–1 and 1–2-1 canals, thorough instrumentation and obturation of one canal theoretically could compensate inadequate treatment of the other, as apical sealing can be achieved through either of the two canals. However, the presence of organic tissue in the non-treated canal may compromise the long-term prognosis.

Table 4 shows the incidence of accessory canals is 17.9%, and most of them are apical delta. The percentage is higher than that reported by Wolf et al. [2] (13.6%) in the German population, but lower than 40% (central incisors) and 26% (lateral incisors) in the Brazialian population [3]. Compared with the main canals, accessory canals are narrower in dimension (*D* = 192 ± 107 μm; *d* = 131 ± 53 μm), and 92.3% of them were located within the apical 3 mm. Apicoectomy with a 3 mm apical resection can remove most of the apical delta and infected tissue, which can hardly be cleaned and shaped by conventional techniques. Table 5 suggests root apex resection on mandibular incisors only slightly increases likelihood of exposing the extra lingual canal (at the level of 1, 2, 3 and 4 mm from the apex, and the number of exposed lingual canal was 3, 6, 6 and 7, respectively, and only 3–4 lingual canals were additionally exposed).

The *D/d* ratio reflects degrees of deviation from a round shape, and Table 5 shows the prevalence of long-oval and flattened canals, as well as the *D/d* ratio, increased from the apical 1 mm level to the 4 mm level. This also means that the root apex resection or apical root resorption may be more likely to produce an oval canal shape at the end of the root. Clinicians should be familiar with the distribution of long-oval and flattened canals in the apical portion. Thorough retropreparation and retrofilling of the ribbon zone or flattened area is essential for a predictable periapical healing. The long-oval or flat canals are most frequently identified at the mid-root level, and their incidence reached as high as 46.4% and 32.1%, respectively, and the mean *D/d* ratio reached the highest level of 4.0 for the single canals. This finding agreed with that of Shemesh et al. [15] who demonstrated that in incisors with 1 root canal, long oval canals (*D/d* ≥ 2) were more frequently found in the middle third of the root of mandibular central and lateral incisors in 36.8% and 48.9%, respectively. This may pose challenges on root canal instrumentation and post space preparation, as the clinicians are put in a dilemma between choosing inadequate preparation of the bucco-lingual dimension or over-instrumentation of the mesio-distal dimension. Majority of the NiTi-rotary systems have a fixed shape and taper, and prone to create a space representing their shape [36]. Therefore, instrumentation of the oval or flattened canal may leave large amounts of debris and uninstrumented areas at the recess or the isthmus areas, or lead to over-instrumentation at the mesial or distal wall. Sufficient canal irrigation or deeper penetration with small files using ultrasonics is recommended to remove tissues from the irregular space of the complex root canal system. Recently, XP-endo Shaper (FKG Dentaire, La Chaux-deFonds, Switzerland), an adaptive single-file NiTi rotary system, was reported as a favorable technique for preparation of the oval or ribbon-shaped (flattened) canal. It is made of a proprietary alloy (MaxWire, Martensite-Austenite Electropolish Flex, FKG Dentaire), which allow the file changing its shape according to the temperature [37, 38]. When cooled, the file stands straight in its martensitic phase, while submitted to body temperature, it changes to its austenitic phase assuming a snake shape that can apply minimal stress to the dentin wall and adapt easily to canal irregularities. The self-adjusting file system has also been reported to possess advantages in increasing the efficiency of irrigating solutions on the smear layer and debris removal, especially at the irregular space of the canal [39, 40]. In regard to the double canal systems, the separated two canals were more likely located at the middle third of the root (as type 1–2-1 was the most frequent variation form). The lingual canals are relatively narrower and rounder than the buccal canals (the mean value of *D*, *d*, and *D/d* is usually lower in the lingual canals than in the buccal canals). Clinically, 1 mm short of radiographic apex can be regarded as the apical endpoint of the working length [41]. In the current study, the canal size at this level (1 mm from apex) was: *D* = 392 ± 169 μm, *d* = 226 ± 70 μm for the single canals; *D* = 308 ± 10 μm, *d* = 232 ± 63 μm for the buccal canals and *D* = 239 ± 25 μm, *d* = 203 ± 22 μm for the lingual canals (Table 3). Weine [42] recommended to enlarge the apical foramen 2 sizes more after having determined the diameter of the original physiological foramen. Therefore, for mandibular incisors, the master file (MAF) size we recommended should be at least ISO 45 (the diameter of the ISO instrument tip was 0.45 mm). However, clinicians also should be aware that the size of the apical file cannot be accurately determined as the middle or coronal segment of the canal is narrow (due to aging or canal calcification) or severely curved.

In clinical view, the roots of mandibular incisors are often straight or slightly curved towards the distal side [1]. While in the proximal view, the root apex usually curved buccally, and measurement of canal curvatures in double-canaled mandibular incisors demonstrated that double curvatures were very common, and the incidence was 50% (8/16) for the buccal canals and 60% (9/15) for the lingual canals. Moreover, severe curvatures are more frequently detected in a canal (either for the buccal, or for the lingual canal) with double curvatures than those with a single curvature (Table 6, Fig. 4D). Zheng et al. [42] measured the root canal curvatures of 299 extracted mandibular incisors based on a radiographic investigation, and found that the canal curvatures were mainly seen in the proximal view (84.3%), with the mean degrees of 9.99 ± 5.84 for the primary curvatures, and 12.10 ± 6.35 for the secondary curvatures. However, Zheng and co-workers [42] had not measured the curvatures of the double-canaled system. The measured data in this study (Table 6) were much greater than those reported by Zheng et al. [42], and the mean degrees of primary curvatures reached 21.5 ± 7.1 (the buccal canals) and 30.1 ± 9.2 (the lingual canals), and the mean degrees of secondary curvatures reached 27.0 ± 11.4 (the buccal canals) and 30.5 ± 12.5 (the lingual canals), suggesting the clinicians may confront an even greater challenge for root canal instrumentation. Previous studies demonstrated that the traditional stainless-steel files may produce a series of procedural errors (such as ledging, zipping and canal transportation) during instrumentation of a curved canal; Niti-rotary system can not only improve the preparation efficacy, but also reduced the incidence of procedural errors and other compliances due to their enhanced flexibility [43]. However, in the double-curved canals, mental fatigue may accumulate at the divergence and convergence sites of the 2 canals, where the acute angle of the canal axis is always corresponding to stress concentration of the instruments. These sites are risk points for occurrence of instrument breakage. To reduce the degrees of canal curvatures during root canal instrumentation, Logani et al. [45] recommended a labial access opening to more consistently provide a straight line access to the apex, as the traditional lingual approach is not ideal for locate the extra lingual canal or may leave untreated dentin areas at the lingual aspect of a flattened canal. However, the current study indicates that the labial access is unfavorable for straight-line access to the buccal canal, especially when it exhibits double curvatures. Several other scholars demonstrated that in anterior teeth, preparing access cavities at the incisal edge facilitated visibility throughout the endodontic treatment, provided straight-line access to the root canal and preserved pericervical dentine [46, 47]. However, basing on in vitro studies on extracted mandibular incisors, Rover et al. [48] and Dos Santos Miranda et al. [49] found that access cavities at/near the incisal edge did not impact on root canal preparation nor resistance to fracture of extracted mandibular incisors as compared to the traditional lingual access cavity design. The preparation of the double canal system and long-oval canal in mandibular incisors with currently available techniques remain challenging [49], and further studies are essential to develop more precise, less invasive, and individualized treatment strategy. The microguided endodontics basing on 3D printing technique may be a potential avenue for a novel therapeutic approach [50, 51].

### Limitations

This study has several limitations. First, majority of the sample teeth were collected from the older people, as the mean age of the subjects was 58 years. The small size of the main or accessory canals was associated with a lower value of *D* and *d*, and the data is more suitable for guiding the endodontic treatment of the older patients rather than the young subjects. Second, although micro-CT has demonstrated its unique value in endodontic researches, it has the unavoidable demerits of higher radiation exposure and limited scanning scope, and can only be applied to in vitro studies. To verify our findings, further in vivo studies basing on CBCT technique are warranted with a larger sample size and different age groups, as a high number of teeth guarantees a solid statistical analysis. Finally, Vertucci’s classification system can’t cover all complex root canal forms, and the inter-canal communications may cause confusion over root canal classification; more accurate classification systems (such as the system of Ahmed et al. [51] involving the use of codes for tooth numbering, root number, and canal configuration) can be applied in our future studies. However, selecting appropriate classification system is always determined by the purpose of the study, and researchers are constantly confronted with a dilemma of selecting accuracy or convenience.

## Conclusions

In conclusion, double-canaled mandibular incisors were not uncommon in the Chinese population, and the type 1–2-1 canal is the most frequent non-single canal type. Gender and age did not significantly impact the occurrence of double canals either in mandibular central, or mandibular lateral incisors. Long-oval and flattened canals are frequently detected in the mid-root portion, as well as 1–4 mm from the apical level. Severe canal curvatures were frequently detected in the double-canal systems, especially in those canals with double curvatures.

## Data Availability

All the datasets used and analyzed during the current study are available from the corresponding author on reasonable request.

## Abbreviations

CBCT: Cone-beam computed tomography
micro-CT: Micro-computed tomography
3D: Three-dimensional
CEJ: Cemento-enamel junction
ICC: Intraclass correlation coefficient
*D*: Long diameter
d: Short diameter
